# Part II: After you accept that faculty position …

**DOI:** 10.1111/cns.14390

**Published:** 2023-08-07

**Authors:** Rehana K. Leak, Devika Soundara‐Manickam, Xiaoming Hu, Jun Chen, Khalid M. Kamal, Wilson S. Meng

**Affiliations:** ^1^ Graduate School of Pharmaceutical Sciences School of Pharmacy, Duquesne University Pittsburgh Pennsylvania USA; ^2^ Department of Neurology University of Pittsburgh School of Medicine Pittsburgh Pennsylvania USA; ^3^ Department of Pharmaceutical Systems and Policy School of Pharmacy, West Virginia University Morgantown West Virginia USA

**Keywords:** academia, career, interview, laboratory, research, tenure

## 1 INTRODUCTION

The objective of Part II of this *CNSNT* editorial series is to help you hit the ground running upon arrival at your new position. As far as possible, you will want to sail across the hurdles in your way, not barely clear them and risk failure. Here, we help you construct a fulfilling career path while managing to stay in the race.

The most crucial tasks you need to perform as early as possible are, (1) reading the literature, (2) designing your experiments, and (3) writing new grant proposals. Ensure that the scientific questions you intend to answer are fundamentally important. This is *the most critical* decision you will make. The more people find your ideas significant and exciting, the easier will be your acquisition of funding.

In the beginning, you can diversify risk by initiating more than two projects, because your favored hypotheses may not be supported by preliminary data. It is not advisable to start researching an entirely new field as a junior investigator. Rather, focus on what you can do well—with utmost rigor—to succeed as quickly as possible and be correct in your interpretations. In the long run, the citations will not matter as much as the accuracy of your observations. Remember that false positives (Type I statistical errors) are more commonly encountered in biomedical research than false negatives (Type II statistical errors).

Plan to start a variety of projects, to be able to publish short‐term, low‐risk projects well before the tenure and/or promotion application deadlines, and then follow up with more impactful work to show a positive slope in your publication trajectory. If the publication rate follows a negative slope, this can also be justified to the committee, if you were shooting for quality over quantity.

In many cases, it is imperative to submit grant proposals in that first year, as reviewers are more forgiving of newly appointed faculty. Keep close track of the “early‐stage” investigator status for grant applications. You will need to strategize how much time you spend on research versus teaching at the beginning. Bear in mind that grants, publications, and student/peer evaluations of teaching will be weighed more heavily toward the end of the tenure clock—and that a rising trajectory is more impressive than a falling trajectory.

Intelligent use of social media can help you disseminate your research to a wider community and raise the possibility of seminar invitations and research collaborations. However, be careful what you write or endorse. Frequent social media postings may give others the (mis)impression that you are not busy. Committee members can also conduct online searches for anything tied to your name.

## 2 IMMEDIATELY BEFORE ARRIVAL

After you sign the contract and before you arrive on site, you can begin to order items, if your budget overseers agree to put away the packages in your allotted space. Interview and hire at least one member of your crew before arrival. Your laboratory should be busy opening packages and filling cabinets and refrigerators as early as possible—while you are busy reading, writing, and generating new ideas.

Learn to delegate activities that require fewer skills. However, be wary of rushing into hiring laboratory members. For example, only agree to mentor students if they seem like a good fit with your expectations for independent scientific thinking and work ethic. Otherwise, it may be worth waiting for the next admissions cycle. It is quite difficult to judge these variables from a Zoom or in‐person interview, and it can take months or years of working together before you know the character of a person in the workplace.

Before you arrive, you can also request syllabi for courses you will teach. If possible, try to get notes/handouts from prior versions of the course to glean the depth and breadth of lecture content. Try to prepare at least some of the first lectures in advance of your arrival, to have sufficient time to focus on getting the laboratory running when you are on site. If you have time, submit the Institutional Animal Care and Use (IACUC) protocols for approval before your arrival, to avoid delays in initiating animal work.

## 3 RUN THE LABORATORY LIKE A SMALL, EFFICIENT BUSINESS OPERATION

To save grant and startup monies, you can find vendors outside of the scientific manufacturer category for at least some items. The outdoors/camping sections of box stores sell many goods that are useful in the laboratory, including protective eyewear, first‐aid kits, toolkits, etc. Hemostats available in fishing sections of box stores can be used for rodent cardiac perfusions. Fishing and camping sections also sell cheap ice buckets with handles. You can use 70% isopropyl alcohol from the grocery store for decontaminating tissue culture spaces. Pharmacies sell various types of inexpensive gloves. Many surgical instruments are less expensive from veterinary suppliers. Almost *anything* will be cheaper than from a “scientific” vendor.

As stated earlier, it is helpful if your offer letter states how long the startup funds will remain available. If you begin to get grants quickly upon arrival, you could then save some of your startup for a future dry spell in funding. It is, of course, difficult to project how much money you will need, but you can enlist assistance from colleagues who work in your field. After 6–12 months on the job, it will be easier to calculate monthly spending rates per trainee and project ahead.

## 4 MANAGE SERVICE PRUDENTLY

Service is an underappreciated variable at most institutions but can also generate unconscious biases for or against your application. You should therefore negotiate with your chairperson to serve on impactful committees. Serving on grant proposal study sections, editorial boards, dissertation/theses committees, and society/conference committees can sharpen your research capacities, mentoring skills, and scientific reputation. Service must be strategically managed in this manner, even if you do not seek leadership opportunities.

Do not agree to reviewing dozens of papers or grants at the expense of the quality of your own work (and your sleep); it is easy to become overburdened by service responsibilities. Review others' work fairly and keep your antennae up for malicious gossip or ill‐informed guesswork on who the reviewer is. You can quickly check social media for emotional outbursts on the part of the authors, but a better gauge of the quality of your reviews is a continued stream of requests by journal editors and funding bodies.

## 5 CONTINUOUSLY UPDATE THE EVIDENCE

To streamline the submission process at the time of your application for retention, tenure, or promotion, keep copies of the evidence of your accomplishments in one electronic file folder, with subfolders organized for scholarship, teaching, and service. Otherwise, collecting all the evidence in one swoop may be overwhelming. When in doubt, simply save the evidence as an electronic document, no matter how unimportant it seems at first. After all, you do not have to submit all the evidence you collect.

A copy of the faculty handbook should always be close at hand, as motivation to refocus your energies on “stuff that counts” and to drop things that will not matter in the long run—although they seem important now. This implies that you direct your research laboratory in a way that does not force you to continually put out fires. If the latter is the case, you may want to manage your personnel better by using performance evaluations. Be sure to document underperformance and problematic behaviors from the beginning.

Put together a list of senior investigators in your field as potential external reviewers of your tenure package. You can invite some as speakers or co‐chair sessions at conferences, review manuscripts for them, and send them your new papers.

In the application, do not forget to explain why your work is important to a general audience, as reviewers may not have your scientific training. Have a layperson and your mentors read the package to ensure it is clear and compelling. Be sure to send it to readers who will critique it thoroughly.

## 6 CONCLUSION

**FIGURE 1:**
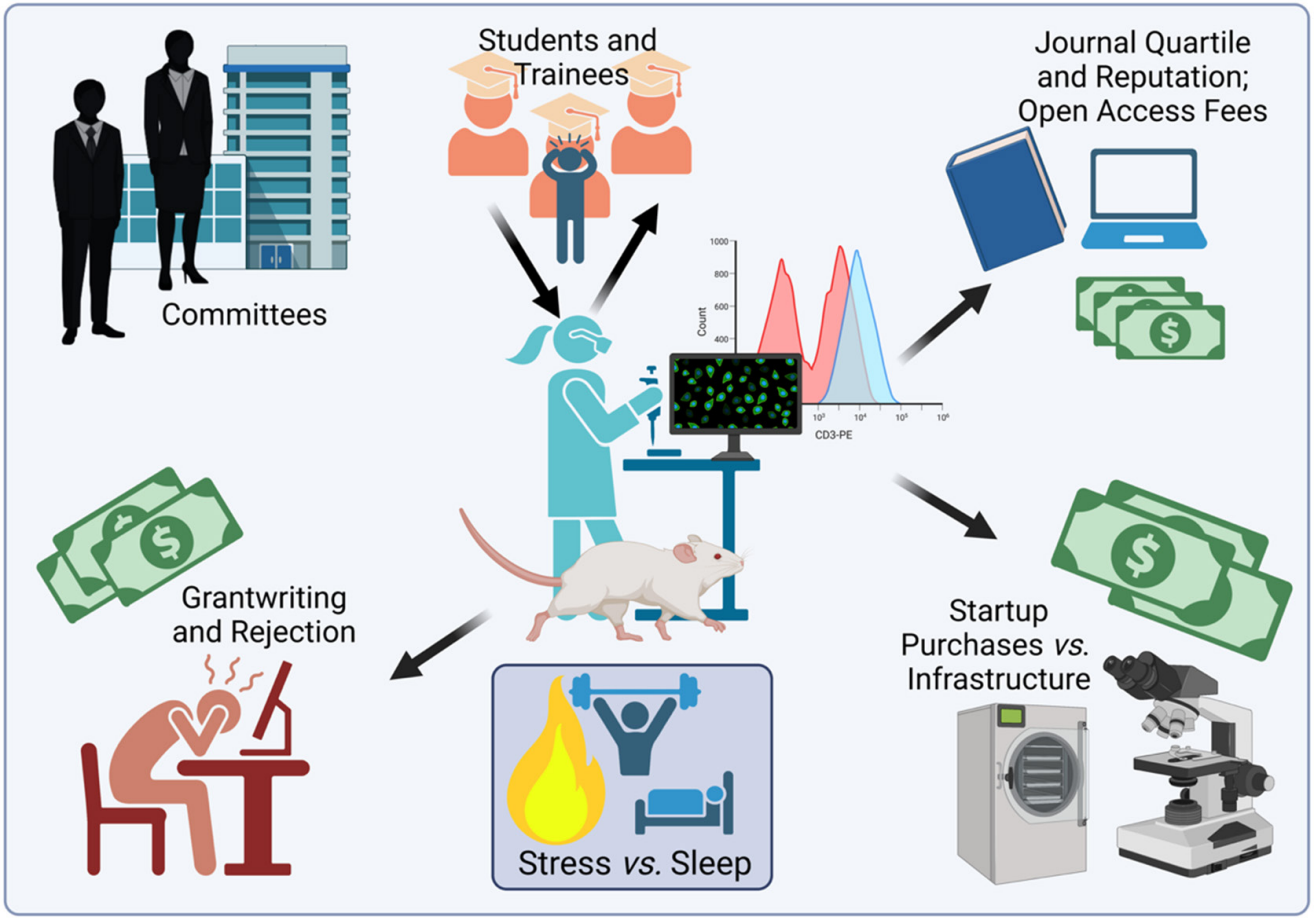
Factors in the race for retention, tenure, and promotion. Created in BioRender®.

Your research career is an endurance contest that will strain the limits of your mental and physical stamina. Do not exhaust all your energetic and financial resources in the beginning. It is true that you will be swamped with a heavy workload, including managing people and finances, which most scientists are wholly unprepared for. However, you will think more clearly, be less forgetful, and regulate your emotions better if you prioritize sleep when you go home (Figure 1). Provide the right incentives (e.g., first authorship) to also motivate your laboratory members to be diligent, without foregoing the rest they also need, or the quality of their work will suffer.

Finally, you may have to steel yourself against the barbs of anonymous review and brace yourself for potentially devastating rejections. Remember that you have joined the faculty body at an academic or research institution because you admire the power of the scientific method, and you have fun doing experiments and thinking about what the data imply. You will need to hold on to that positive perspective, pick yourself up, dust the rejections off, and focus like a laser beam on the next goals. Take comfort in the fact that you become more efficient and productive as you mature, which will help you enjoy your work all the more.

## CONFLICT OF INTEREST STATEMENT

The authors have no competing interests to declare. No approvals were gathered for this work.

## Data Availability

No data were collected during the creation of this report, but any further questions can be emailed to leakr@duq.edu

